# Supplementary material for: Single Cell Transcriptomic and Chromatin Profiles Suggest Layer Vb Is the Only Layer With Shared Excitatory Cell Types in the Medial and Lateral Entorhinal Cortex
Source: Front Neural Circuits. 2022 Jan 26;15:806154. doi: 10.3389/fncir.2021.806154 (PMC8826650; doi:10.3389/fncir.2021.806154)
Supplement: Supplementary file 14 [file Data_Sheet_1.ZIP › Exc_vs_Inh_Motifs/homerResults/motif1.similar.html]

motif1

## Information for motif1

T
G
A
C
T
C
A
G
G
A
T
C
C
G
T
A
C
A
T
G
C
T
A
G
C
G
A
T
C
T
A
G
A
G
T
C
C
A
T
G
  
Reverse Opposite:  

G
T
A
C
T
C
A
G
G
A
T
C
G
C
T
A
G
A
T
C
G
T
A
C
G
C
A
T
C
T
A
G
A
G
T
C
A
C
T
G
  

|  |  |
| --- | --- |
| p-value: | 1e-3486 |
| log p-value: | -8.029e+03 |
| Information Content per bp: | 1.616 |
| Number of Target Sequences with motif | 17554.0 |
| Percentage of Target Sequences with motif | 41.61% |
| Number of Background Sequences with motif | 1712.3 |
| Percentage of Background Sequences with motif | 15.75% |
| Average Position of motif in Targets | 288.1 +/- 232.0bp |
| Average Position of motif in Background | 343.3 +/- 254.8bp |
| Strand Bias (log2 ratio + to - strand density) | 0.0 |
| Multiplicity (# of sites on avg that occur together) | 1.40 |
| Motif File: | file (matrix) reverse opposite |

### Similar de novo motifs found

|  |  |  |  |  |  |  |  |
| --- | --- | --- | --- | --- | --- | --- | --- |
| Rank | Match Score | Redundant Motif | P-value | log P-value | % of Targets | % of Background | Motif file |
| 1 | 0.981 | G A T C C T A G A G T C C G T A A C T G A C T G A C G T A C T G | 1e-3333 | -7674.776635 | 46.24% | 19.52% | motif file (matrix) |
| 2 | 0.976 | A T C G A G T C C T G A A G T C C G T A A G T C A G T C A C G T A C T G A G T C C T G A C A G T | 1e-3304 | -7609.273174 | 45.99% | 19.43% | motif file (matrix) |
| 3 | 0.809 | T C A G G A T C C G T A A C T G A C T G A C G T C T G A C A G T | 1e-2307 | -5312.470027 | 75.64% | 51.22% | motif file (matrix) |
| 4 | 0.816 | C G T A C A T G T C A G G A C T C T G A A C T G C T A G C G A T C T A G C A T G | 1e-2091 | -4816.213443 | 66.16% | 42.43% | motif file (matrix) |
| 5 | 0.842 | T C A G G T A C C T G A A C T G A C T G G A C T C T A G A C T G T C A G C G A T A C T G A C G T | 1e-997 | -2295.945591 | 33.61% | 19.58% | motif file (matrix) |
| 6 | 0.634 | T C G A T C G A G T A C C G T A A C T G C G T A C G A T A C T G A T C G G A C T C G A T C A T G | 1e-880 | -2028.543092 | 43.84% | 29.21% | motif file (matrix) |
| 7 | 0.654 | A G T C G T A C C G T A C G A T A T G C A C G T A C T G A G C T | 1e-700 | -1612.916813 | 26.14% | 15.39% | motif file (matrix) |
| 8 | 0.687 | A C G T T G A C G C T A G A T C C G T A G T A C T G A C G A C T G A C T C G T A | 1e-452 | -1043.014502 | 59.13% | 48.06% | motif file (matrix) |
| 9 | 0.725 | T C G A C G T A C G T A A G T C A T G C A C G T A C T G A C T G | 1e-368 | -847.931011 | 22.02% | 14.54% | motif file (matrix) |
| 10 | 0.629 | A C T G C T G A T C A G C T A G A C G T C T G A C A G T C T A G | 1e-338 | -780.376165 | 27.27% | 19.36% | motif file (matrix) |
| 11 | 0.738 | G A T C A G T C G C T A C A T G C A T G G A C T G C A T G A C T | 1e-329 | -758.178260 | 66.05% | 56.80% | motif file (matrix) |
| 12 | 0.658 | C T G A C G T A A G T C C G T A A G T C A G T C A C G T A G T C | 1e-135 | -311.610464 | 6.99% | 4.33% | motif file (matrix) |
